# Ultrasound-guided cable-free 13-gauge vacuum-assisted biopsy of non-mass breast lesions

**DOI:** 10.1371/journal.pone.0179182

**Published:** 2017-06-19

**Authors:** Jiwoon Seo, Sun Mi Kim, Mijung Jang, Bo La Yun, Soo Hyun Lee, Eun-Kyu Kim, Eunyoung Kang, So Yeon Park, Woo Kyung Moon, Hye Young Choi, Bohyoung Kim

**Affiliations:** 1 Department of Radiology, Seoul National University Bundang Hospital, Seoul National University College of Medicine, Seoul, Republic of Korea; 2 Department of Surgery, Seoul National University Bundang Hospital, Gyeonggi-do, Republic of Korea; 3 Department of Pathology, Seoul National University Bundang Hospital, Gyeonggi-do, Republic of Korea; 4 Department of Radiology, Seoul National University College of Medicine, Seoul National University Hospital, Seoul, Republic of Korea; 5 Department of Radiology, Gyeongsang National University Hospital, Jinju-si, Republic of Korea; 6 Division of Biomedical Engineering, Hankuk University of Foreign Studies, Oedae-ro 81, Mohyeon-myeon, Cheoin-gu, Yongin-si, Gyeonggi-do, Korea; Fu Jen Catholic University, TAIWAN

## Abstract

**Purpose:**

To compare the outcomes of ultrasound-guided core biopsy for non-mass breast lesions by the novel 13-gauge cable-free vacuum-assisted biopsy (VAB) and by the conventional 14-gauge semi-automated core needle biopsy (CCNB).

**Materials and methods:**

Our institutional review board approved this prospective study, and all patients provided written informed consent. Among 1840 ultrasound-guided percutaneous biopsies performed from August 2013 to December 2014, 145 non-mass breast lesions with suspicious microcalcifications on mammography or corresponding magnetic resonance imaging finding were subjected to 13-gauge VAB or 14-gauge CCNB. We evaluated the technical success rates, average specimen numbers, and tissue sampling time. We also compared the results of percutaneous biopsy and final surgical pathologic diagnosis to analyze the rates of diagnostic upgrade or downgrade.

**Results:**

Ultrasound-guided VAB successfully targeted and sampled all lesions, whereas CCNB failed to demonstrate calcification in four (10.3%) breast lesions with microcalcification on specimen mammography. The mean sampling time were 238.6 and 170.6 seconds for VAB and CCNB, respectively. No major complications were observed with either method. Ductal carcinoma in situ (DCIS) and atypical ductal hyperplasia (ADH) lesions were more frequently upgraded after CCNB (8/23 and 3/5, respectively) than after VAB (2/26 and 0/4, respectively *P* = 0.028).

**Conclusion:**

Non-mass breast lesions were successfully and accurately biopsied using cable-free VAB. The underestimation rate of ultrasound-detected non-mass lesion was significantly lower with VAB than with CCNB.

**Trial registration:**

CRiS KCT0002267.

## Introduction

Rapid and accurate diagnosis of suspected breast cancer lesions is highly important for both women with cancer and those without significant breast issues who require reassurance. Currently, percutaneous core needle biopsy, a rapid, cost-effective, highly sensitive, and highly specific method that facilitates definitive diagnoses and provides prognostic information, is considered standard practice, thus preventing the need for open surgical biopsy or frozen-section analysis [1]. Ultrasound (US) is the preferred first-line imaging modality for breast biopsy, and US-guided core biopsy is a cost-effective, rapid method that facilitates definitive diagnosis and provides prognostic information, thus allowing prompt decisions about future treatment options.

The 14-gauge (G) spring-loaded core biopsy device is most commonly used for breast lesion sampling. Although this device features advantages such as a minimum setup requirement, low cost, and lack of spatial requirements, its disadvantages include the requirement for a unique insertion and removal of the needle for each biopsy, leading to difficulties with constant targeting and increasing procedure durations. In addition, some of the obtained samples are of low quality because of fragmentation or dry tapping. In contrast, vacuum-assisted biopsy (VAB) overcomes the above mentioned disadvantages by offering consistently high sample quality and a single insertion step. Accordingly, VAB is more reliable with respect to decreasing the rates of false negatives and underestimation and causing fewer complications [2, 3]. However, VAB also has limitations such as the requirements for an increased setup time, a dedicated assisting technologist, and space for large equipment.

The recently introduced cable-free VAB devices provide the advantages of both spring-loaded core biopsy devices (e.g., minimal setup and space requirements) and VAB (consistently high sample quality, single insertion step). These devices comprise a non-firing probe with a 13-G needle aperture and collecting cup [4]. Biopsy samples are cut at the aperture and transported to the collecting cup by vacuum suction.

A non-mass lesion (NML) on US does not meet the current criteria of a mass according to the Breast Imaging Reporting and Data System (BI-RADS); therefore, it is not included in the BI-RADS system lexicon. However, we often encounter NMLs, which appear as hypoechoic areas that correlate with microcalcifications on mammography or suspicious non-mass enhancement (NME) on magnetic resonance imaging (MRI). These NMLs can reflect a wide spectrum of pathologic changes such as fibrocystic changes, fibrosis, mastitis, papilloma, ductal carcinoma in situ (DCIS), invasive ductal cancer, and invasive lobular cancer [5].

Although a recent study reported preliminary results regarding the potential advantage of a cable-free handheld VAB device to diagnose DCIS from NMLs [6], no published studies have described a comparison of the outcomes of this new device with those of 14-G conventional core needle biopsy (CCNB). Therefore, we aimed to compare the outcomes of US-guided core needle biopsy by 14-G semi-automated CCNB and by 13-G cable-free VAB.

## Materials and methods

### Patients

This prospective study was approved by the institutional review board of the Seoul National University Bundang Hospital, and written informed consent was obtained from all patients. This study was registered with the Clinical Research Information Service (registration no. KCT0002267). However, this study was registered after enrolling participants because this was not randomized clinical trial therefore we didn’t aware of trial registration. The authors confirm that all ongoing and related trials for this drug/intervention are registered.

**Fig 1:**
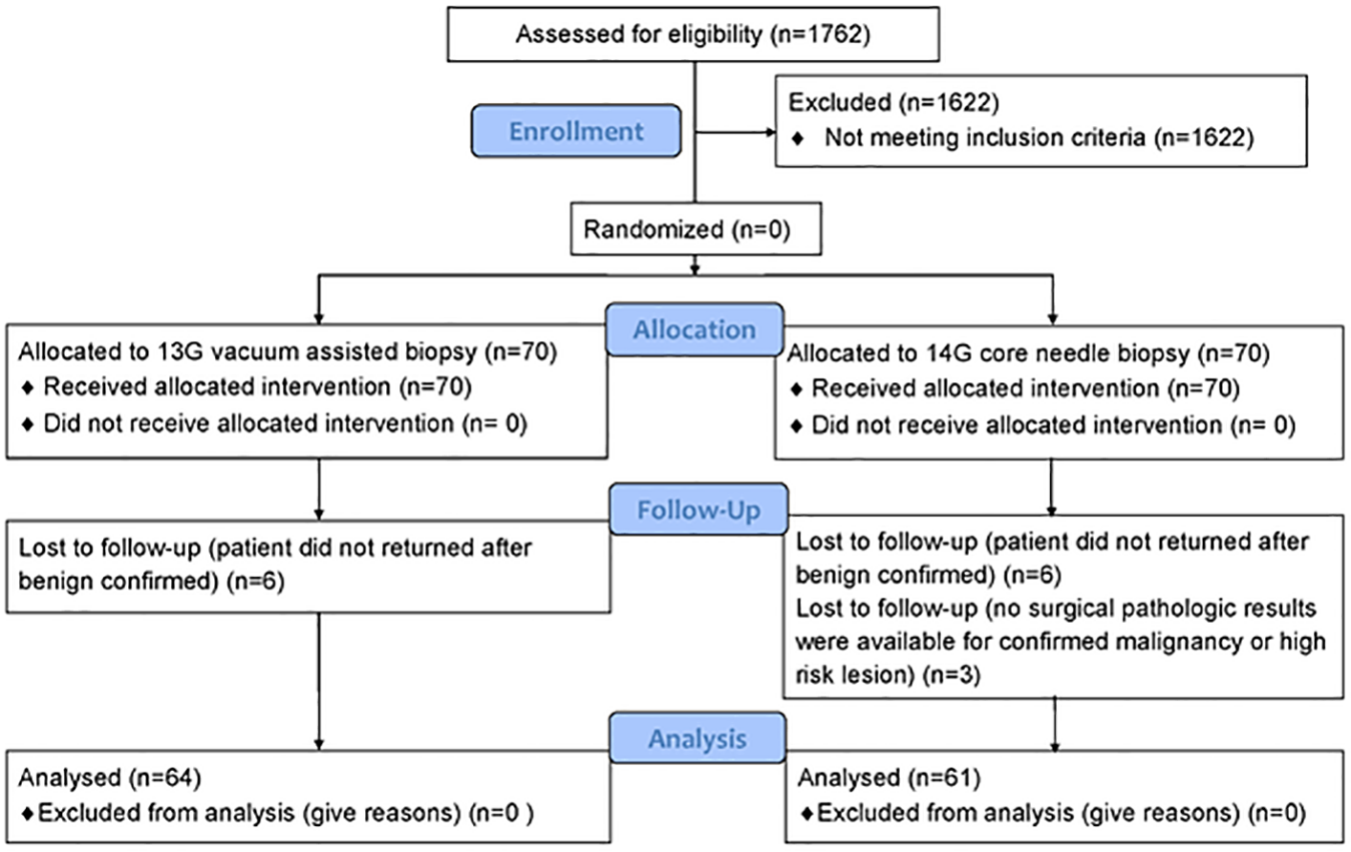
CONSORT flow chart.

All women with US-detected NMLs who were referred to our breast radiology unit for US-guided percutaneous core needle biopsy from August 2013 through December 2014 were considered eligible for our study. During the study period, a total of 1840 US-guided percutaneous core needle biopsies were performed in 1762 patients (mean age, 46.26 years; range, 19–92 years). The inclusion criteria for breast NMLs were as follows: NMLs classified as BI-RADS category 4 or 5 with suspicious microcalcifications on US that correlated with a lesion previously detected via mammography, or US-correlated lesion detected via breast MRI. The exclusion criteria were a lack of available follow-up images of benign confirmed lesions and a lack of available surgical pathologic results of confirmed malignancies or high-risk lesions. A flowchart of the patient selection process is shown in Fig 1.

### Sample size

The study initially aimed to investigate the sensitivity and specificity and the rates of histological underestimation and side effects of 13-G VAB. The sample size was calculated to be 216 patients when the power was set at 80%, the level of significance at 5%, and the drop-out rate at 5%. Therefore, all lesions detected by US-guided breast biopsy were initially included. However, a previous retrospective study reported that wireless VAB was more advantageous for detecting NMLs than for detecting mass lesions [6].On the basis of this report, it was more appropriate that the subjects were limited to those with NML and comparison of underestimation rates; therefore, the sample size was recalculated. According to the superiority test, the superiority margin was set at 15%. The number of samples to achieve the power of 80% at a significance level of <5% was calculated at 76 patients per arm.

### Imaging evaluation

Mammography was performed using dedicated equipment (Senographe; GE Medical Systems, Milwaukee, WI, USA) and a Lorad/Hologic Selenia Dimension full-field digital mammography System (Lorad/Hologic, Danbury, CT, USA). US was performed using high-resolution US equipment (iU22; Philips Healthcare, Bothell, WA, USA). Diagnostic MRI was performed using a 3-T closed-bore MRI system (Achieva, Philips Medical Systems, Best, The Netherlands) with a dedicated phased-array bilateral breast coil (MRI devices, Wurzburg, Germany) and gadolinium contrast. Real-time sonographic assessment was performed by one of three radiologists with 5, 9, and 13 years of breast imaging experience. Before biopsy, each lesion was assigned a final BI-RADS assessment category according to mammography and US findings.

### Biopsy technique

All biopsies were performed after the patients received an explanation of the risks and benefits, and provided written informed consent. One of three dedicated breast radiologists performed biopsies via the 14-G semi-automated CCNB with a STERICUT^®^ device (TSK Laboratory, Tochigi, Japan) or via the new method with a 13-G cable-free VAB device (Mammotome Elite^®:^ Devicor Medical Products, Cincinnati, OH, USA) according to the patient’s preference after patients were provided with explanations regarding both types of biopsy needles. Following local anesthesia and skin incision, a 14-G CCNB or 13-G VAB needle was placed at the target lesion under US guidance. A minimum of three cores were obtained for each lesion. For cases with microcalcifications, specimen mammography was obtained to ensure biopsy of the areas of microcalcification. If the specimen mammography did not include microcalcifications, an additional round of biopsies was performed.

Immediate post-procedural complications were determined by the radiologists. Immediate biopsy site hematoma was evaluated using US, and US-guided compression was performed in the case of particularly in patient with active bleeding lesion. Delayed complications were identified by the referring surgeons 1 week after the biopsy when the patient returned for the biopsy result. Immediately after the procedure, patients were interviewed about the pain experienced during the biopsy and were asked to indicate this pain intensity on an 11-point Numerical Pain Rating Scale ranging from 0 (none) to 10 (extreme, worst possible pain).

### Follow-up, data collection, and analysis

All histopathologic findings were reviewed by a single pathologist. Clinical, radiologic, and pathologic data were collected for statistical analysis. For cases with a pathologic diagnosis of malignancy or borderline lesions such as atypical ductal hyperplasia (ADH), surgical resection was recommended. For cases with discordant radiologic and pathologic findings, further surgical biopsy was recommended. For cases in which both the imaging and histologic diagnoses were benign, follow-up US, mammography, or MRI at 6-month intervals was recommended. The end date for data collection was August 2016. We collected data of the average number of specimen samples, biopsy sampling range, and tissue sampling time. To evaluate the latter parameter, we recorded the elapsed time from needle insertion to removal. The final surgical pathologic result was compared with the obtained core needle biopsy results to determine the rate of diagnostic upgrade or downgrade. The patient pain scores and post-biopsy complications mentioned above were also compared between the two groups.

### Statistical analysis

The chi-square test and Fisher’s exact test, were used to compare the existence of symptoms, BI-RADS category, lesion type, final pathologic result, DCIS underestimation and lesion type, ADH underestimation and lesion type, and complication rates, between the VAB and CCNB groups. [R5-2] The Student's t-test or Mann-Whitney test was performed to compare the age of the patients, size, number of biopsy, biopsy duration, and score of pain between the VAB and CCNB groups.

The statistical analysis was conducted using MedCalc for Windows, version 14.8.1 (MedCalc Software, Mariakerke, Belgium) and SPSS (version 24.0). A *P* value of <0.05 was considered to indicate a statistically significant difference.

## Results

### Patients and target lesions

**Fig 2:**
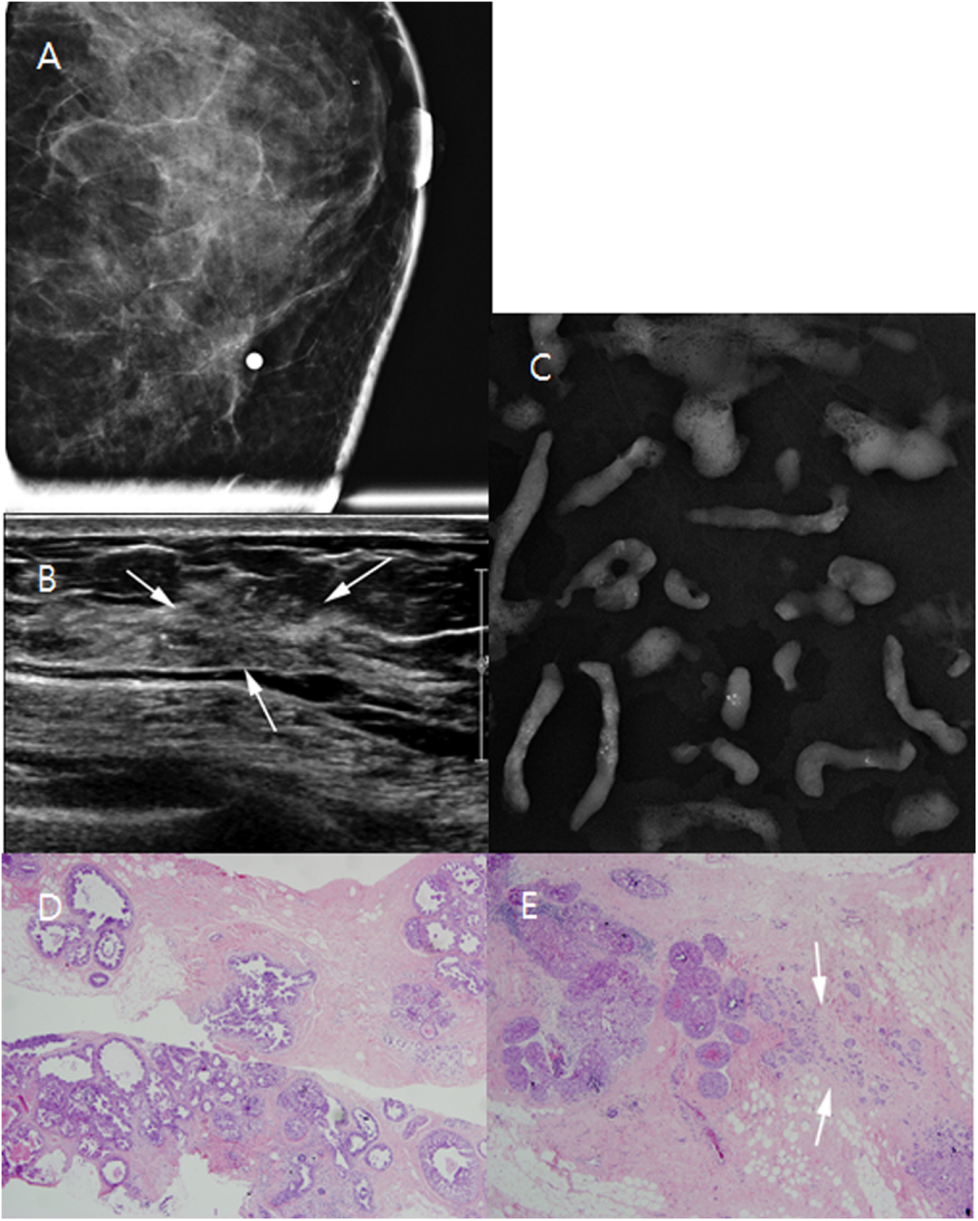
An abnormality detected via screening mammography in a 47-year-old woman. (A) Left craniocaudal magnification and compression views reveal regional amorphous microcalcifications that correlate with the ultrasound (US)-detected lesion (mammography skin marking). (B) US shows microcalcification in a heterogeneously hypoechoic area (arrows). (C) Specimen mammography indicates a large amount of microcalcification. (D) Photomicrography of 13-gauge vacuum-assisted biopsy (VAB; original magnification, ×40; hematoxylin and eosin [H&E] stain) reveals a ductal carcinoma in situ (DCIS) with involvement in multiple ductal spaces. (E) After total mastectomy, the final diagnosis was upgraded to invasive ductal carcinoma with a 0.3-cm invasive focus (arrows) and 7.7-cm area of DCIS (original magnification, ×40; [H&E] stain).

**Fig 3:**
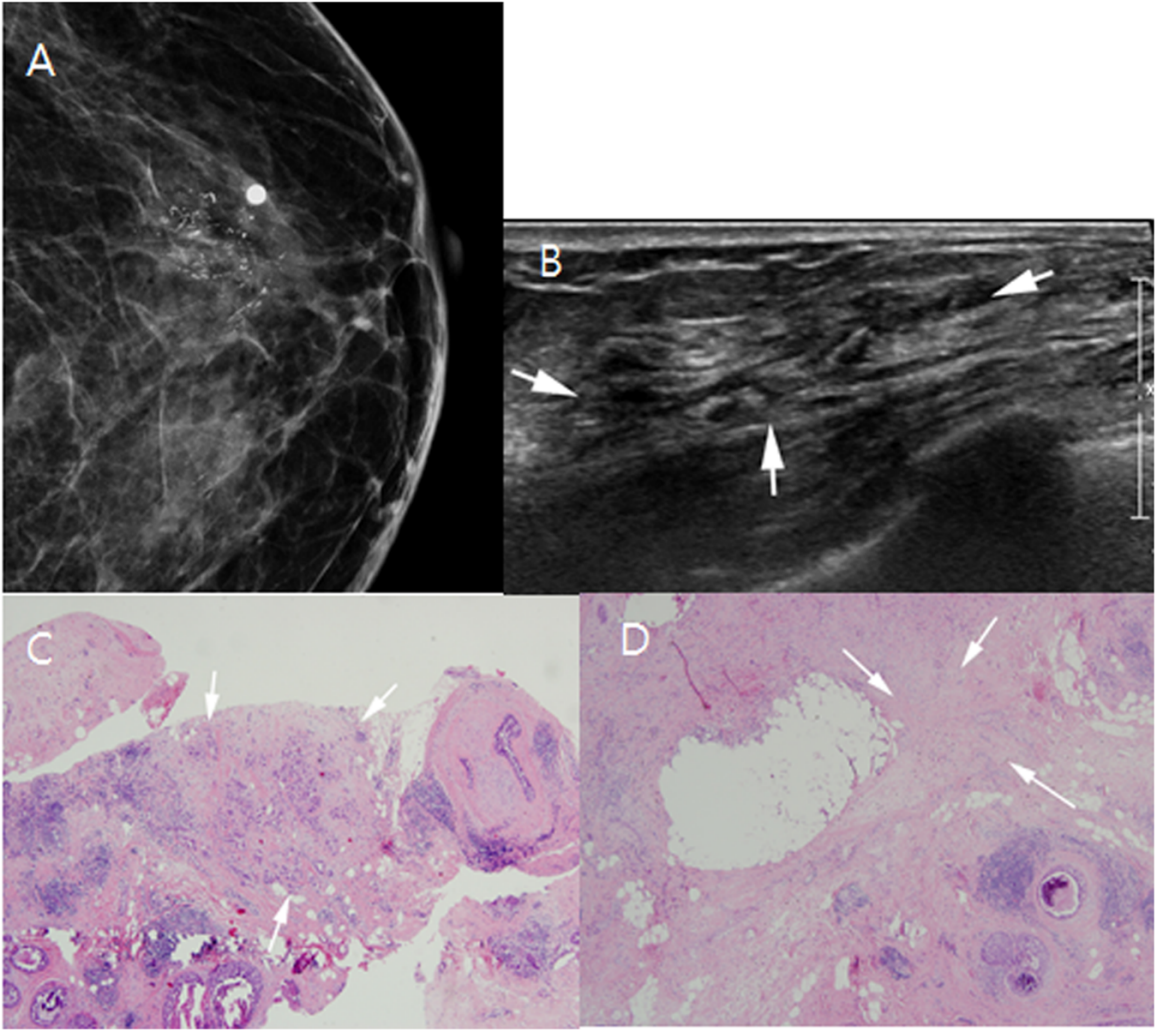
An abnormality detected during screening mammography in a 69-year-old asymptomatic woman. (A) Left craniocaudal magnification and compression views show segmental pleomorphic microcalcifications that correlate with the ultrasound (US)-detected lesion (mammography skin marking). (B) US shows ductal dilatation with internal microcalcification (arrows). (C) Photomicrography of a 13-gauge vacuum-assisted biopsy (original magnification, ×40; hematoxylin and eosin [H&E] stain) shows tumor cell infiltration into the stroma; this case was confirmed as an invasive ductal carcinoma (arrows). (D) Only ductal carcinoma in situ (DCIS; 5.6-cm extent) remained in the mastectomy specimen. Photomicrography (original magnification, ×40; H&E stain) shows a post-biopsy scar area (arrows) in the DCIS background.

**Fig 4:**
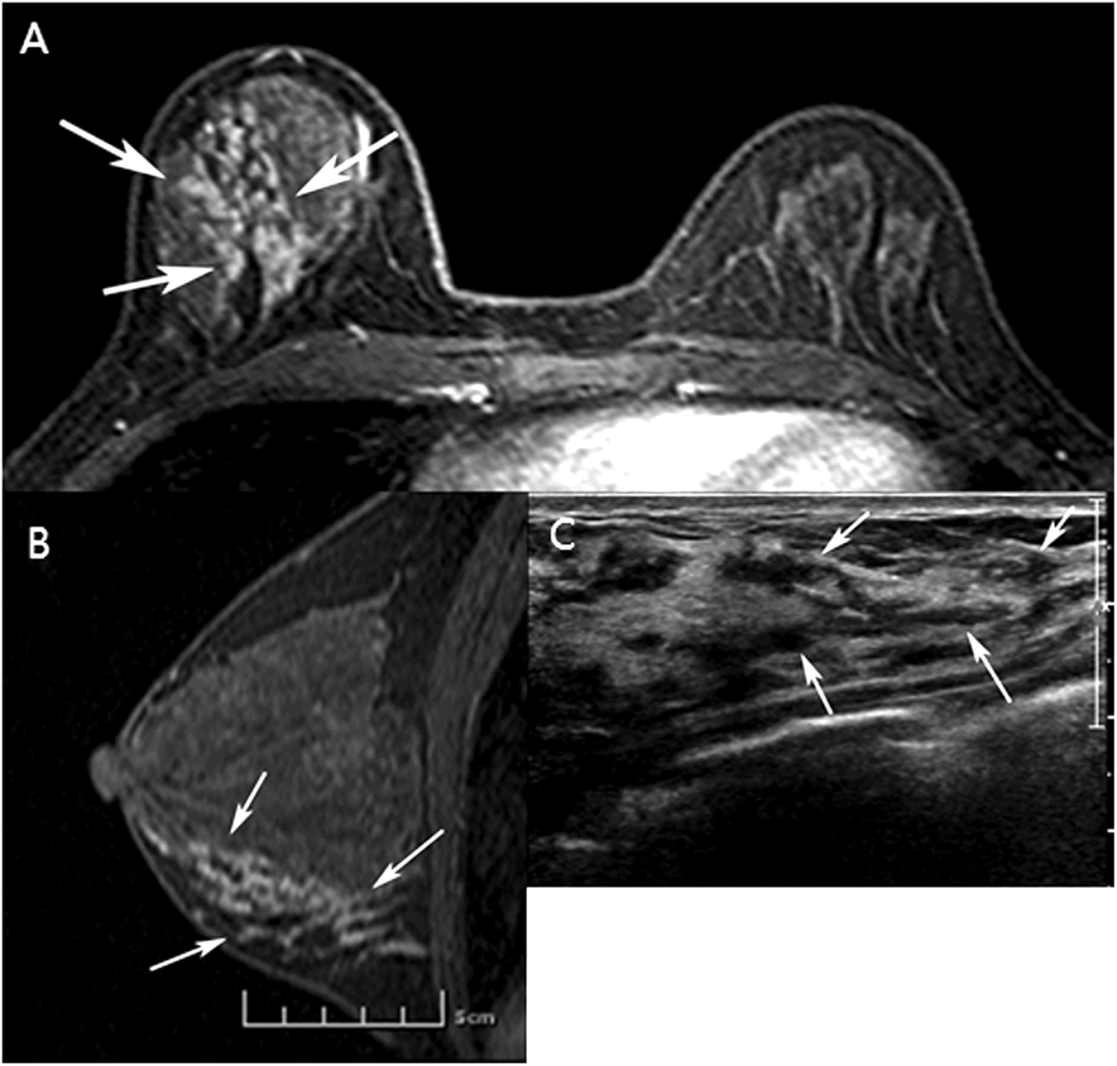
Invasive ductal carcinoma in the upper inner quadrant of the right breast of a 39-year-old woman. (A, B) Axial (A) and reformatted sagittal (B) T1-weighted magnetic resonance imaging (MRI; T1WI) with early post-gadolinium enhancement show segmental non-mass enhancement in the lower portion of the right breast (arrows). (C) Ultrasound of the non-mass lesion demonstrates a correlation with MRI findings (arrows). This non-mass lesion was confirmed as ductal carcinoma in situ via 13-gauge vacuum-assisted biopsy and surgery.

**Fig 5:**
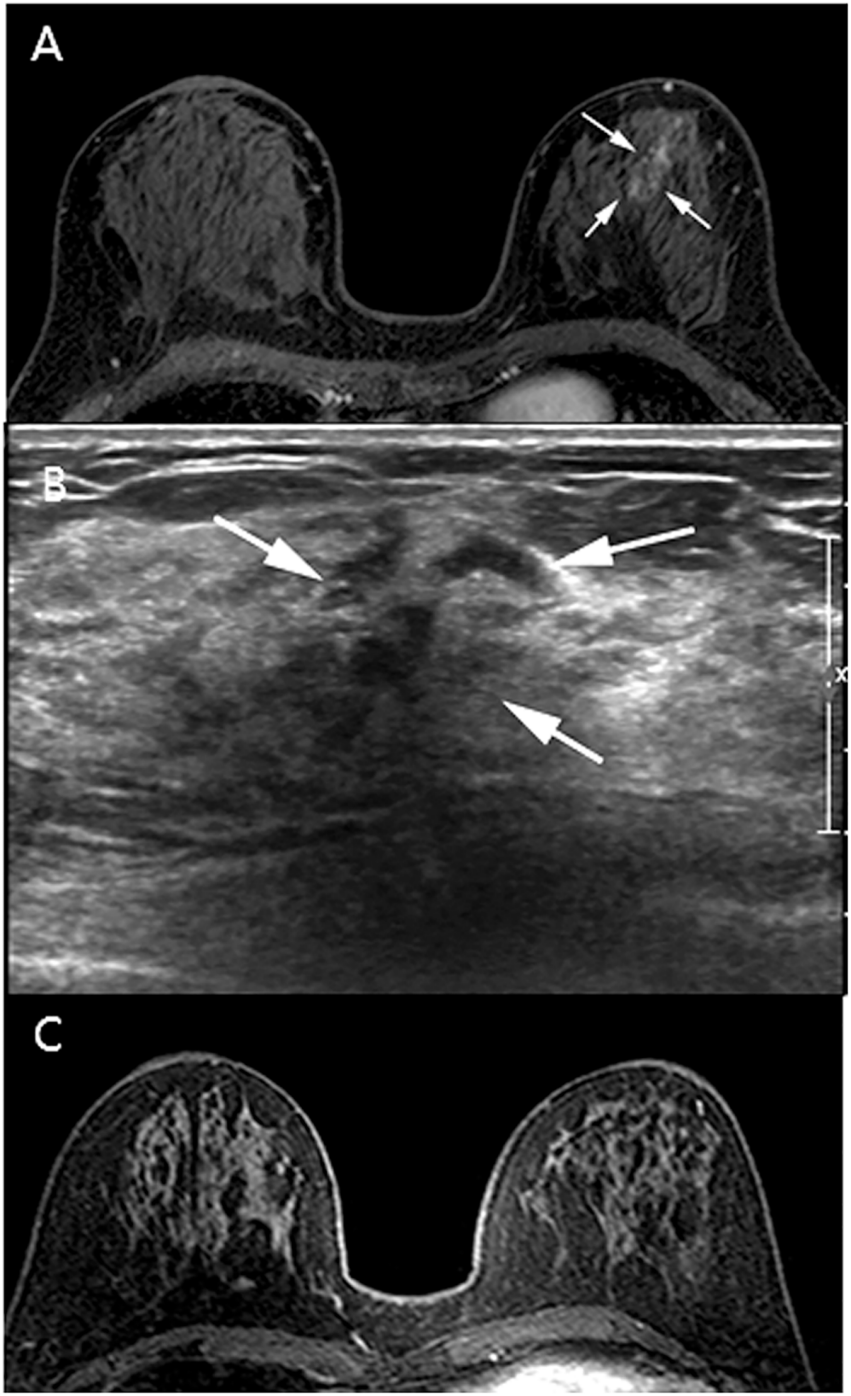
A 49-year-old woman with a history of breast-conserving surgery for right breast cancer. (A) Axial T1WI with early post-gadolinium enhancement shows focal non-mass enhancement in the upper portion of the left breast (arrows). (B) Ultrasound of the NML shows a correlation with MRI findings (arrows). This non-mass lesion was confirmed as a fibrocystic change via 13-gauge VAB. (C) Follow-up MRI showed disappearance of focal non-mass enhancement.

Among the 1840 US-guided percutaneous core needle biopsies performed during the study period, 160 (8.7%) NMLs were detected in 140 patients; which were BI-RADS category 4 or 5 lesion, the lesions with suspicious microcalcification (Figs 2 and 3) or those with US-MRI-correlated findings (Figs 4 and 5). Twelve lesions in 12 patients were excluded because follow-up images of benign confirmed lesions were unavailable, and three lesions in three patients were excluded because no surgical pathologic results were available for confirmed malignancies or high-risk lesions. Finally, 145 lesions in 125 patients were included in this study. Of those, 64 patients with 68 lesions agreed to undergo biopsy with 13-G VAB, and 61 patients with 77 lesions who did not agreed underwent biopsy with 14-G CCNB. The target lesion characteristics and patient demographics are described in Table 1. There were no significant intergroup differences in patient age, lesion size, the number of symptomatic patients, BI-RADS category, lesion type, and rate of malignancy between the VAB and CCNB groups (Table 1).

**Table 1:** Characteristics of patients and lesions.

Variable	13-G VAB (n = 68)	%	14-G CCNB (N = 77)	%	Total	%	*p* value*
**Patient age (years)** **(Student t-test)**							
**Mean ± SD**	47.5 ± 10.04		48.1 ± 11.3				0.70
**Range**	29–72		28–83				
**Lesion size (cm)** **(Mann-Whitney test)**							
**Mean ± SD**	2.15 ± 1.75		1.97 ± 1.91				0.19
**Range**	0.3–9		0.4–8.6				
**Symptom(Chi-square test)**	8/68		8/77				0.79
**BI-RADS category** **(Fisher’s exact test)**							
**4a**	42	61.8	42	54.5	84	57.9	0.40
**4b**	16	23.5	8	10.4	24	16.6	0.04
**4c**	2	2.9	9	11.7	11	7.6	0.06
**5**	8	11.8	18	23.4	26	17.9	0.08
**Lesion type(Chi-square test)**							0.09
**Microcalcification**	44	64.7	39	50.6	83	57.3	
**MR-correlated lesion**	24	35.3	38	49.4	62	42.7	
**Final pathology result(Chi-square test)**							0.92
**Benign**	35	51.5	39	50.6	74	51	
**Malignancy**	33	48.5	38	49.4	71	49	

Note: SD, standard deviation; BI-RADS, Breast Imaging Reporting and Data System; 13-G VAB, 13-gauge vacuum-assisted biopsy; 14-G CCNB, 14-gauge core needle biopsy; MR, magnetic resonance.

*Two-sided *p* value.

### Technical success rate and pathologic results

US-guided VAB successfully targeted and sampled NMLs with and without calcifications. For breast lesions with microcalcifications, US-guided VAB successfully targeted 40 (91%) lesions during the first biopsy attempt and the remaining four (9%) during the second attempt. Specimen mammography demonstrated that all 44 target lesions had been sufficiently sampled. None of the biopsy procedures required cancelation because of an inability to visualize or reach the target lesions. No radiologic–pathologic discordance was observed.

Although 14-G CCNB also successfully targeted breast lesions without calcifications in a single biopsy attempt, this technique failed to demonstrate calcifications on specimen mammography in four (10.4%) breast lesions with microcalcifications. However, 14-G CCNB successfully targeted 32 (82%) lesions during the first biopsy attempt and three (7.6%) during the second attempt. One case of radiologic–pathologic discordance was observed, and it was confirmed as a benign lesion after subsequent further excision.

The biopsy results are shown in Table 2. All malignancies (n = 33) and high-risk lesions (n = 11) identified via US-guided VAB were subjected to surgery. Among the 24 cases of benign lesions identified by US-guided VAB, two cases of sclerosing adenosis were treated surgically. Twenty patients with 22 benign lesions underwent imaging follow-up within 6 months (mean follow-up, 22.6 months; range, 6–28 months). In the 14-G CCNB group, all the patients with lesion confirmed as malignancy (n = 35) and high-risk lesions (n = 11) underwent surgery. One fibrocystic change lesion was confirmed as pseudoangiomatous stromal hyperplasia on subsequent 8-G VAB. Thirty benign lesions were followed up within 6 months (mean follow-up, 21 months; range, 6–30 months).

**Table 2:** Histologic diagnoses.

Malignant	13-G VAB (n = 68)	%	14-G CCNB (N = 77)	%
DCIS	26	79	23	65.7
DCIS with microinvasion	3	9	2	5.7
IDC	3	9	9	25.7
Mucinous	1	3		
ILCA			1	2.9
Total	33		35	
**High-risk lesions**				
FEA	1	9.1	2	18.2
Papillary neoplasm	3	27.25	3	27.25
LCIS	3	27.25	1	9.1
ADH	4	36.4	5	45.45
Total	11		11	
**Benign**				
Fibroadenoma	8	33.3	9	29.05
Fibrocystic change	6	25.0	8	25.8
Adenosis	5	20.8	8	25.8
Inflammation changes	1	4.2	1	3.25
Others	4	16.7	5	16.1
Total	24		31	

Note: 13-G VAB, 13-gauge vacuum-assisted biopsy; 14-G CCNB, 14-gauge core needle biopsy; DCIS, ductal carcinoma in situ; IDC, invasive ductal cancer; ILCA, invasive lobular cancer; FEA, flat epithelial atypia; LCIS, lobular carcinoma in situ; ADH, atypical ductal hyperplasia

Two of 26 cases of DCIS were upgraded to invasive ductal cancer (IDC) (Fig 2); however, no cases of ADH identified with VAB were upgraded according to the final surgical pathologic results. In contrast, eight of 23 DCIS cases and three of five ADH cases identified via 14-G CCNB were upgraded after surgery (Tables 3 and 4). Comparatively, statistically significant differences were observed between 13-G VAB and 14-G CCNB in the number of upgrades of DCIS and ADH (2/32 vs. 11/39, *P* = 0.028). One case of IDC and two cases of DCIS with microinvasion did not show invasive foci upon subsequent surgery and VAB (Fig 3), and the surgical specimen of one case of ADH did not contain residual ADH (S1 Fig).

**Table 3:** Underestimation of DCIS.

	13-G VAB (n = 26)	14-G CCNB (n = 23)	*p* value *
**DCIS underestimation (Fisher’s exact test)**	2/26	8/23	0.03
**Lesion size (cm) (Mann-Whitney test)**			
**Mean ± SD**	2.09 ± 1.63	2.74 ± 2.35	0.12
**Range**	0.5–7	0.5–8.2	
**Lesion type(Fisher’s exact test)**			
**Microcalcification**	2/20	7/18	0.06
**MR-correlated lesion**	0/6	1/5	1.00

Note: DCIS, ductal carcinoma in situ; SD, standard deviation; 13-G VAB, 13-gauge vacuum-assisted biopsy; 14-G CCNB, 14-gauge core needle biopsy; MR, magnetic resonance

*Two-sided *p*-value.

**Table 4:** Underestimation of ADH.

	13-G VAB (n = 4)	14-G CCNB (n = 5)	*p* value *
**ADH underestimation** **(Fisher’s exact test)**	0/4	3/5	0.49
**Lesion size (cm) (Mann-Whitney test)**			0.03
**Mean ± SD**	0.7 ± 0.36	2 ± 1.17	
**Range**	0.3–1.1	1–3.9	
**Lesion type(Fisher’s exact test)**			
**Microcalcification**	0/1	0/0	1
**MR-correlated lesion**	0/3	3/5	0.49

Note: ADH, atypical ductal hyperplasia; SD, standard deviation; 13-G VAB, 13-gauge vacuum-assisted biopsy; 14-G CCNB, 14-gauge core needle biopsy; MR, magnetic resonance

*Two-sided *p* value.

### Sampling time and number of specimens

Notably, 13-G VAB required a significantly longer time than 14-G CCNB, with mean total procedure time of 238.6 seconds (range, 90–680 seconds) vs. 170.6 s (range, 109–360 s; Table 5), respectively. The significantly longer 13-G VAB procedure time was largely attributable to the significantly larger number of biopsy samples obtained with 13-G VAB (mean, 8.9; range, 4–18) vs. 14-G CCNB (mean, 5.97; range, 3–10; Table 5). However, despite the increased time and biopsy number, patients reported similar levels of pain with VAB (Table 5).

**Table 5:** Biopsy time, numbers, and pain scores.

	13-G VAB (n = 68)	14-G CCNB (n = 77)	*p* value *
**Time (s) (Mann-Whitney test)**			
**Mean ± SD**	238.6 ± 115.9	170.6 ± 61.8	<0.001
**Range**	90–680	109–360	
**Number(Mann-Whitney test)**			
**Mean ± SD**	8.9 ± 3.5	5.97 ± 1.63	<0.001
**Range**	4–18	3–10	
**Pain score(Mann-Whitney test)**			
**Mean ± SD**	1.41 ± 0.87	1.83 ± 1.46	0.96
**Range**	1–6	1–7	

Note: SD, standard deviation; 13-G VAB, 13-gauge vacuum-assisted biopsy; 14-G CCNB, 14-gauge core needle biopsy

*Two-sided *p* value.

### Complications

Hematomas of <3 cm in size were observed on immediate post-biopsy US in seven patients who underwent VAB and four patients who underwent CCNB (*P* = 0.35). All hematomas decreased in size at OPD visit 1 week after biopsy (S2 Fig). No major complications were observed during biopsies with either technique, and none of the biopsies were interrupted by complications. None of the patients reported severe pain, and none developed hematomas or wound infections requiring treatment.

## Discussion

A NML on US is observed as hypoechoic area, not a definite mass. Microcalcifications are usually poorly identified on US or just observed as echogenic foci in NML. A non-mass enhancing lesions on MRI also might appear as NMLs on US. Histologically, NMLs are often heterogeneous and are frequently confirmed as malignancies such as DCIS or invasive lobular cancer [5, 6]. Therefore, the histologic agreement between US-guided core needle biopsy and surgical diagnosis is significantly lower in cases of NMLs than that of mass lesions [7].

14-G CCNB has been the most common technique for percutaneous breast biopsy for its high sensitivity and positive predictive value for breast lesions and a lower rate of repeated biopsy compared with fine-needle aspiration biopsy, which results from inadequate sampling [8, 9]. Since its introduction in 1995, VAB has become preferred method for certain lesions, such as small clusters of microcalcification, architectural distortions or lesion which requires complete removal, as the larger core specimens and contiguous sampling facilitated by the VAB device yield better retrieval of the target [10, 11]. The European Society of Breast Imaging guideline also noted that VAB (8–11 G) could be used for cases with indeterminate or obviously malignant clusters of microcalcification, with discordant results after 14-G core needle biopsy, and with architectural distortions, as well as to increase the likelihood of detecting invasive foci [2]. The recently introduced 13-G cable-free VAB has been described as handy, with the potential benefit of accurate pathologic diagnosis [6].

In the current study, we evaluated the performance of a newly introduced handheld cable-free 13-G VAB device and compared the outcomes with those achieved with a semi-automated 14-G CCNB device, which represents the standard of care for breast lesions at our center. MRI-correlated NMLs were successfully sampled using both techniques, as were most NML with microcalcifications (approximately 90%) during the first attempt. During the second attempt, all NMLs with microcalcifications were successfully sampled with 13-G VAB, whereas 14-G CCNB sampling failed in four cases (10.4%). Given the previously reported retrieval failure rates of 7.1%–14% with 14-G CCNB and 3%–29% with 11-G or 8-G VAB [12–15], US-guided 13-G VAB appears superior for the retrieval of microcalcifications.

Percutaneous biopsy for the diagnosis of a breast lesion occasionally fails to fully characterize the target lesion, resulting in an underestimation of invasive cancers [16], including ADH, premalignant lesions, and DCIS. Histologically, ADH is defined as at least two duct-connected lines or areas that present with atypical cell changes or a <2-mm area occupied by atypical cells. Other lesions are classified as DCIS [17]. Notably, we observed a significantly lower rate of underestimation with VAB than with CCNB; specifically, our DCIS underestimation rate with 13-G VAB (7.7%) was lower than the rates in previous reports with 11-G VAB (17%–41%). This result is identical to the rates reported by previous studies that used VAB (8-G or 10-G) devices and found that the DCIS underestimation rates decreased with larger acquired tissue samples, which were attributed to decreases in sampling errors [11, 16]. Furthermore, our underestimation rate with CCNB (28.2%) was lower than that of reported by previous studies (39%–67%) [11, 18–21]. Our routine CCNB practice involves the collection and review of >4 non-fragmented samples by a single, dedicated breast pathologist, which may explain our lower underestimation rate.

This lower DCIS underestimation rate was attributed to improved lesion sampling with VAB. Continuous vacuum-assisted sampling can suction air and/or blood away from the biopsy cavity during the procedure, thus solving the problem of introducing air into the biopsy cavity after repeated passes with the core biopsy needle, which could mimic or obscure hyperechoic microcalcification foci [13, 22]. In addition, during VAB, the probe is positioned posterior to the lesion and therefore does not overshadow the lesion. Conversely, in one case of invasive cancer (Fig 3) and two cases of DCIS with microinvasion, no invasive foci were observed during subsequent surgery; one case of ADH showed no residual ADH in the surgical specimen after VAB.

VAB yielded larger sample numbers, which corresponded with a longer sampling time. Our routine core needle biopsy device used a coaxial needle, a factor that might have also decreased the biopsy time. However, multiple samples can be obtained easily via VAB, whereas it is difficult to obtain more than seven samples with CCNB, even with a coaxial needle, because the multiple needle insertions will cause changes that could obscure the lesion.

Our study had several limitations. First, case selection was not randomized, possibly leading to selection bias. Second, in some cases, we only reported a short-term follow-up to describe the initial outcomes of these biopsy devices; however, 6–12 months of additional follow-up would be needed to confirm the benign nature of the lesions. Third, to ensure easier sample acquisition with VAB, we retrieved three to five more samples according to the performer’s discretion, which might have affected the lesion underestimation rate. Fourth, the study population was small; a larger study population and more outcome data are needed to confirm our results.

In conclusion, this new cable-free VAB device successfully and accurately biopsied non-mass breast lesions. The US-detected NML underestimation rate was significantly lower with VAB than with CCNB. Therefore, cable-free VAB could be designated the device of choice for NMLs.

## Supporting information

S1 Fig An abnormality detected via screening mammography in a 42-year-old woman. (A) Left craniocaudal magnification and compression views reveal grouped amorphous microcalcifications (arrow) that correlate with the ultrasound (US)-detected lesion (mammography skin marking).
(B) US guided 13-gauge vacuum-assisted biopsy performed for microcalcifications (arrow) in a heterogeneously hypoechoic area. Pathology revealed atypical ductal hyperplasia.
(C) Specimen mammography indicates a large amount of microcalcification (arrows). Final surgical pathology was no residual atypical ductal hyperplasia.
(TIF)

S2 Fig An abnormality detected on contralateral breast via preoperative breast MRI in a 37-year-old woman. (A) US guided 13-gauge vacuum-assisted biopsy performed for MRI correlating non-mass lesion (arrows). Pathology revealed sclerosing adenosis.
(B) On immediate post biopsy ultrasound shows about 2.5cm hematoma (arrows) developed at biopsy site.
(C) 10days after biopsy, ultrasound shows decreased in size of hematoma (arrows) at biopsy site. Hematoma resolved and non-mass lesion is stable on follow up US and MRI over 2 years.
(TIF)

S1 File Trial study protocol (English). (PDF)

S2 File Trial study protocol (Korean). (PDF)

S3 File TREND statement cheklist. (PDF)
