# Supplementary figures and images for: Ultrasound-guided cable-free 13-gauge vacuum-assisted biopsy of non-mass breast lesions
Source: PLoS One. 2017 Jun 19;12(6):e0179182. doi: 10.1371/journal.pone.0179182 (PMC5476256; doi:10.1371/journal.pone.0179182)

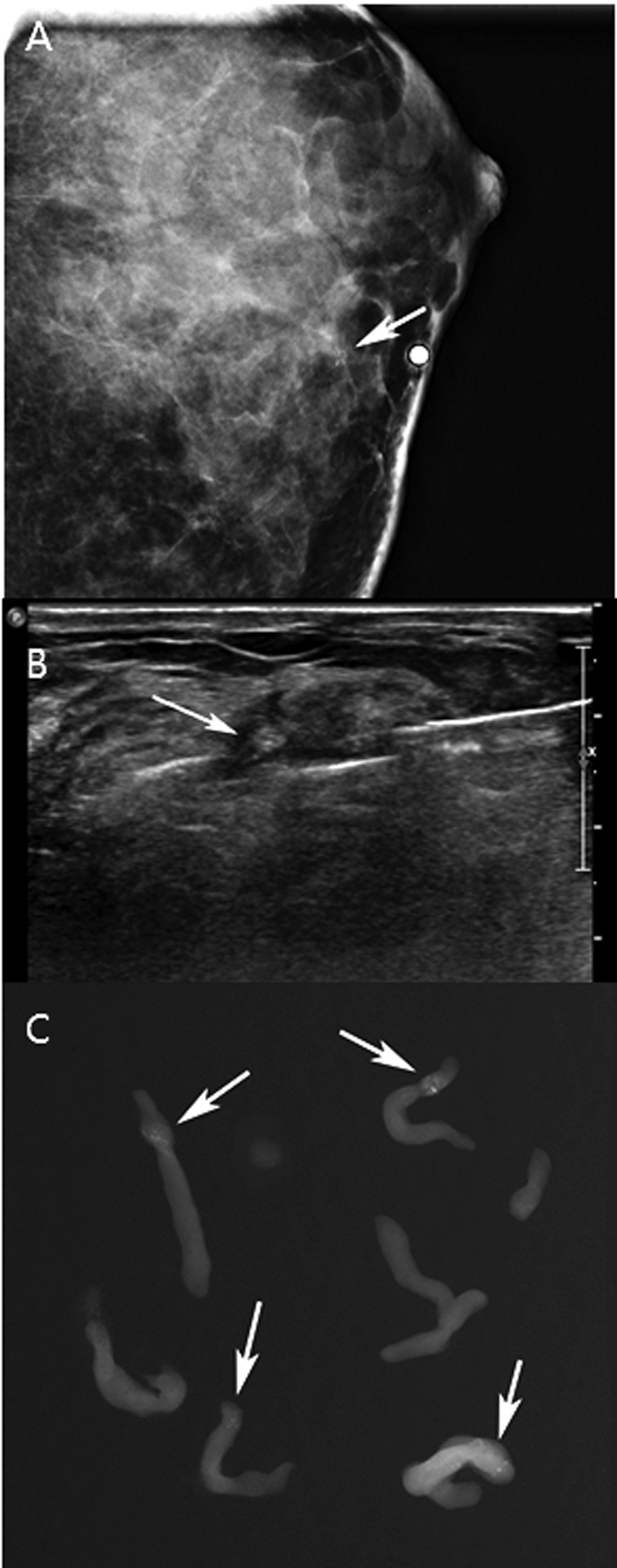

Supplement: S1 Fig — (A) Left craniocaudal magnification and compression views reveal grouped amorphous microcalcifications (arrow) that correlate with the ultrasound (US)-detected lesion (mammography skin marking). (B) US guided 13-gauge vacuum-assisted biopsy performed for microcalcifications (arrow) in a heterogeneously hypoechoic area. Pathology revealed atypical ductal hyperplasia. (C) Specimen mammography indicates a large amount of microcalcification (arrows). Final surgical pathology was no residual atypical ductal hyperplasia. (TIF) [file pone.0179182.s001.tif]

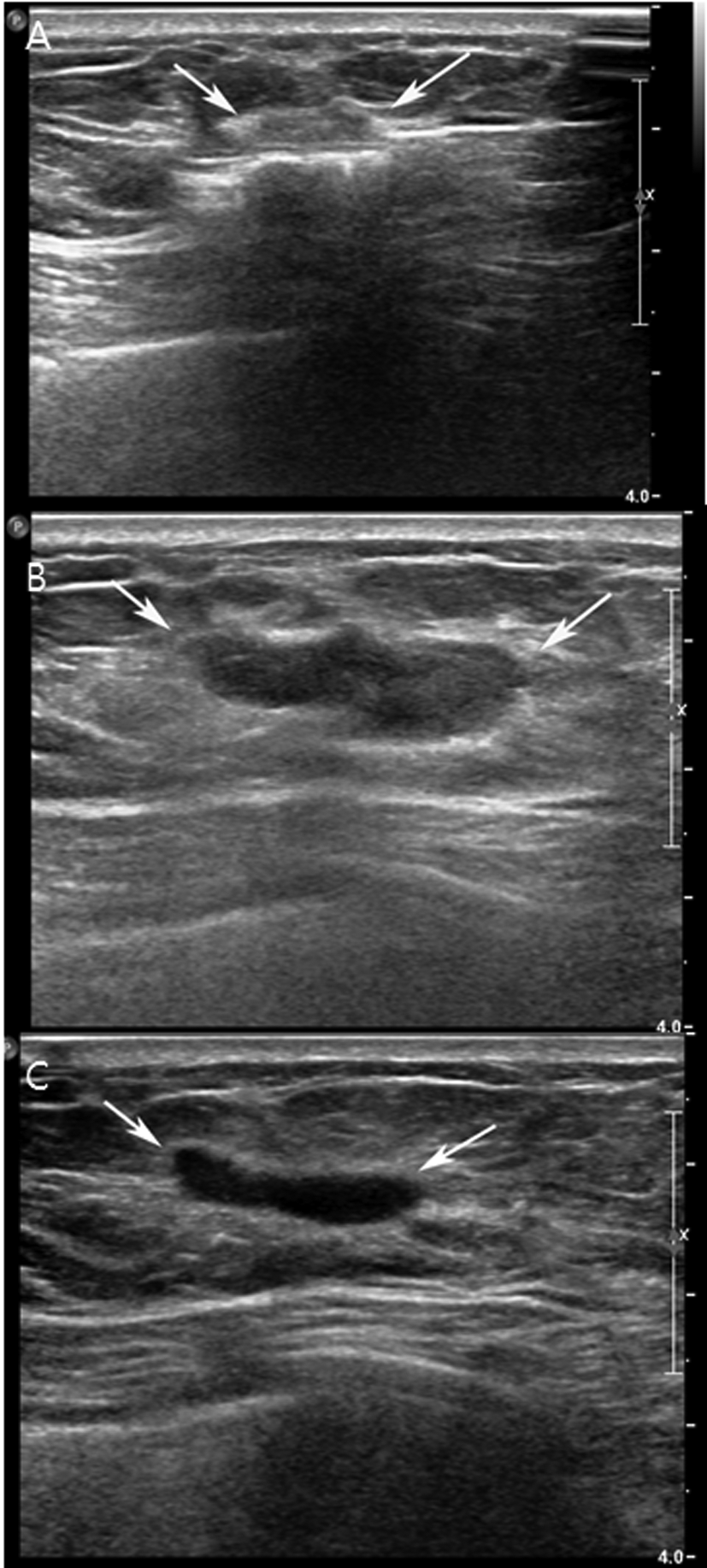

Supplement: S2 Fig — (A) US guided 13-gauge vacuum-assisted biopsy performed for MRI correlating non-mass lesion (arrows). Pathology revealed sclerosing adenosis. (B) On immediate post biopsy ultrasound shows about 2.5cm hematoma (arrows) developed at biopsy site. (C) 10days after biopsy, ultrasound shows decreased in size of hematoma (arrows) at biopsy site. Hematoma resolved and non-mass lesion is stable on follow up US and MRI over 2 years. (TIF) [file pone.0179182.s002.tif]
