# Supplementary material for: Ultrasound-guided cable-free 13-gauge vacuum-assisted biopsy of non-mass breast lesions
Source: PLoS One. 2017 Jun 19;12(6):e0179182. doi: 10.1371/journal.pone.0179182 (PMC5476256; doi:10.1371/journal.pone.0179182)
Supplement: S1 File — (PDF) [file pone.0179182.s003.pdf]

Protocol outline

**Title**	(English) Evaluation of the usefulness of 14G vacuum-assisted breast biopsy in suspicious breast disease.<br>(Korean) Evaluation of the effectiveness of 14-gauge vacuum-assisted breast biopsy in patients suspected of having a breast disease.
**Objective**	The 14G vacuum-assisted breast biopsy collects larger amounts of tissues through a relatively simple process compared to the ultrasound-guided 14-gauge core needle biopsy. It is associated with low histological underestimation and false negative rates. We aim to investigate the indications for, and strengths and weaknesses of the14-gauge vacuum-assisted breast biopsy.
**Research Institution**	Department of Radiology, Seoul National University Bundang Hospital
**Research Director**	Sun Mi Kim, Department of Radiology, Seoul National University Bundang Hospital
**Subjects**	216 patients
**Research Period**	One year from the approval date
**Methods**	Among patients for whom a biopsy was deemed necessary based on their breast ultrasound results, patients who had calcified lesions, intraductal lesions, or lesions less than 2.0cm in size and agreed to participate in this study, all of which vacuum-assisted breast biopsies have known benefits, are included in the present study. Diagnostic accuracy, complication rates, and patients' and clinicians' convenience will be compared to identify appropriate indications for 14G vacuum-assisted breast biopsy and its strengths and weaknesses.
**Expected effects and estimated results**	14G vacuum-assisted breast biopsy is expected to have lower histological underestimation and false negative rates compared to core needle biopsies. If no significant change in the level of discomfort and pain experienced by a patient is observed following a 14G vacuum-assisted biopsy, the biopsy may be expected to prevent additional unnecessary procedures and increase diagnostic accuracy, ultimately increasing patients' trust on clinicians.

1. Title

English: Evaluation of the usefulness of 14G vacuum-assisted breast biopsy in suspicious breast disease

Korean: Evaluation of the effectiveness of 14-gauge vacuum-assisted breast biopsy.

2. Name and Address of the Research Institution

Department of Radiology, Seoul National University Bundang Hospital

82 Gumi-ro 173 beo, Bundang-gu, Seongnam-si, Gyeonggi-do, South Korea

3. Research Director and

3.1 Research director

Sun Mi, Kim, Associate Professor, Department of Radiology, Seoul National University Bundang Hospital

3.2 Research manager

Bora, Yoon, Assistant Professor, Department of Radiology, Seoul National University Bundang Hospital

3.3 Co-researchers

Mijung, Jang, Assistant Professor, Department of Radiology, Seoul National University Bundang Hospital

Hye-Shin, Ahn, Radiologist, Department of Radiology, Seoul National University Bundang Hospital

Eun-Hee, Yang, Researcher, Department of Radiology, Seoul National University Bundang Hospital

4. Expected length of the research period

One year from the IRB approval

5. Methods

5.1 Introduction

1) Objective

Vacuum-assisted 14G breast biopsy is generally expected to have lower histological underestimation and false negative rates, and collects larger amounts of tissues compared to ultrasound-guided 14G needle breast biopsy. We assessed the diagnostic accuracy, complication rates, and patients' and clinicians' convenience with the use of 14G vacuum-assisted breast biopsy to identify indications for, and strengths and weaknesses of 14G vacuum-assisted breast biopsy.

2) Research background and needs

<u>1. Ultrasound-guided breast biopsy</u>

Nonpalpable lesions found on mammograms or breast ultrasounds exhibit nodularity or calcification. Since it is

difficult to differentiate breast cancer from benign tumors by using medical images alone, a biopsy is performed. An ultrasound-guided breast biopsy is less invasive than surgical biopsies and has high diagnostic accuracy of 95%. Patients are exposed to smaller amounts of exposure during an ultrasound-guided breast biopsy than during a stereotatic breast biopsy, and can undergo the procedure in the comfortable, supine position. Clinicians can also check needle locations in real time during the procedure, which only requires a short amount of time.

2. False negative and histological underestimation rates of 14G ultrasound-guided needle biopsy

As mentioned earlier, false negatives are a major drawback of the ultrasound-guided breast biopsy. False negatives refer to when lesions diagnosed as benign are later diagnosed as cancer after an operation. The false negative rates are reported to be 0~3.6% (mean 1.8%) with the 14G needle biopsy. False negative rates are known to be especially high when micro-calcified lesions, masses contain solid and fluid components, small masses that are less than 5mm in size, or papillomas are present. In addition, lesions initially diagnosed as atypical ductal hyperplasia during a biopsy that are later diagnosed as breast cancer after the final operation, or lesions initially diagnosed as ductal carcinoma that are later diagnosed as invasive breast cancer, can pose the problem of histological underestimation. When atypical ductal hyperplasia is diagnosed in a percutaneous biopsy, a histological examination is performed around the area affected by the lesion. Ductal carcinoma accompanied by epithelial cell proliferation is called atypical ductal hyperplasia if the lesion is less than 2mm in size, and ductal carcinoma if the lesion is greater than 2mm in size. Therefore, when lesions are partially atypical or invasive, underestimation of the collected tissue can result.

3. Effectiveness of vacuum-assisted breast biopsy

The vacuum-assisted breast biopsy utilizes the principles of vacuums to obtain tissues in which lesions are present. After a needle is inserted into a tissue, a vacuum is used to aspirate the tissue into the needle. Then, the tissue is cut with a cutting tube and moved to a storage space at the back of the needle by using the vacuum’s sound pressure to be collected. The vacuum-assisted breast biopsy does not require any needle to be inserted for tissue collection unlike needle biopsies. Once a needle is inserted into a suspicious mass, it does not have to be removed from the breast tissue, and can allow the tissue to be collected repeatedly for up to 15-20 times. This method can increase the diagnostic accuracy while decreasing the rate of repeated biopsy. Needles are available in varying thicknesses of 14G, 11G, and 8G. The 11G needles have sensitivity of 98-100% and specificity of 100%.

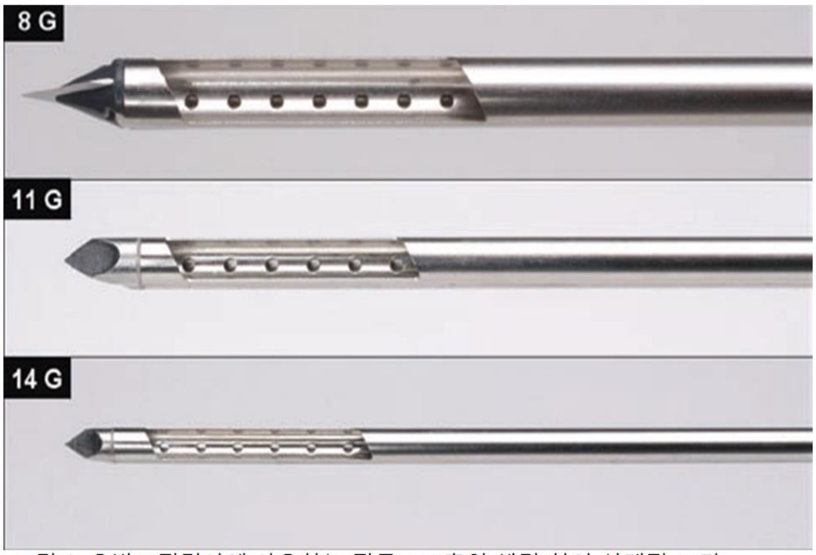

그림 1. 유방조직검사에 사용하는 진공보조흡인 생검 침의 상대적 크기

Image translation: Figure 1. Relative sizes of needles used during a vacuum-assisted biopsy of breast tissues.

The rate of histological underestimation is reported to be lower for atypical ductal hyperplasia and ductal carcinoma when a vacuum-powered 11G needle was used compared to when a needle biopsy is performed. This is because during a single biopsy, a 14G core needle can collect 17mg of tissue, while 8G, 11G, and 14G vacuum-powered needles can collect 300mg, 100mg, and 34mg of tissue, respectively. In other words, vacuum-powered needles can collect twice as much tissue as regular biopsy needles in a single biopsy even when they are of the same gauge. The rate of underestimation of atypical ductal hyperplasia is 16% (95% CI 12-20%), and of ductal carcinoma is 11% for the vacuum-assisted breast biopsy. On the contrary, needle biopsies have high rates of underestimation of 40% (95% Cl 26-56%) for atypical ductal hyperplasia, and of 15% (95% Cl 8-26%) for ductal carcinoma. However, no data regarding the comparison of the diagnostic performance, and postoperative side effects and discomfort between 14G vacuum-powered needles and 14G core needles are available.

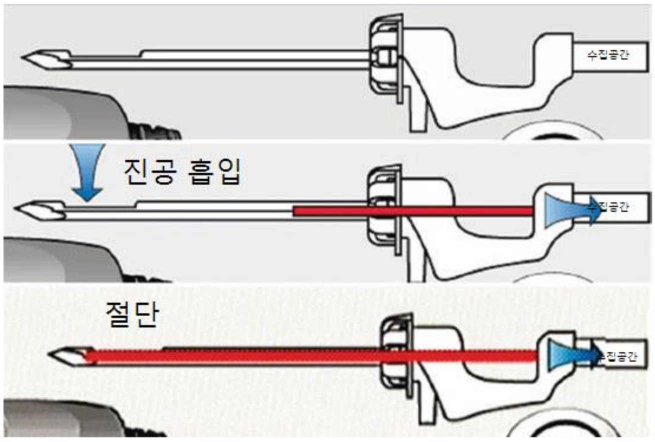

그림 2. 진공보조흡인 생검 침의 조직 채취법

Image translation:

- 진공흡입 = vacuum aspiration
- 절단 = cut
- 그림 2. 진공보조흡인 생검침의 조직 채취법 : Method of tissue collection using a vacuum-powered biopsy needle

5.2 Inclusion and Exclusion Criteria

● Inclusion criteria: Among patients for whom a biopsy was deemed necessary based on breast ultrasound results, those who consented to participating in the present study, and who were expected to have low rates of false negatives or histological underestimation after undergoing a vacuum-assisted breast biopsy were included (patients with lesions exhibiting calcification, intraductal lesions, lesions less than 2.0cm in size, lesions containing solid and fluid components).

● Exclusion criteria: Patients who show contraindications for biopsy (Patients who previously experienced side effects of local anesthesia, or who are at high risk of bleeding), patients who did not consent to participating in the study.

5.3 Sample size calculation

● Although no similar study has been conducted previously, we referred to a prospective study on histological underestimation of papilloma with the use of 11G vacuum-assisted breast biopsy, and another that calculated the sensitivity or specificity of a 11G vacuum-assisted biopsy, in the determination of a patient sample size. The present study has the nature of a pilot study, and was aimed at investigating the sensitivity and specificity of a 11G vacuum breast biopsy, and its rates of histological underestimation and side effects. The sample size was calculated to be 216 patients when the power was set at 80%, the level of significance at 5%, and the drop-out rate at 5%.

5.4 Observation and clinical test targets, and methods of observation

●Obtaining medical images

We visualized lesions in real time by performing an ultrasound prior to a biopsy, and described them according to the Breast Imaging Reporting and Data system of the American College of Radiology.

● **Method of ultrasound-guided vacuum-assisted breast biopsy**

A) Method of examination

* After sterilizing the area round the lesion and the skin onto which the needle is to be placed, a surgical drape was placed on top of the same area.

*** Anesthetize the skin with 1-2ml of 1% lidocaine.**

*** Make an incision on the skin by using a #11 blade.**

*** Anesthetize the route through which a needle will enter the area around the lesion with 4-5ml of 1% lidocaine.**

*** Insert a needle into the breast through the skin incision, and place it near the lesion under ultrasound guidance.**

*** Perform a biopsy.**

*** Pressure the skin for 5-20 minutes to prevent bleeding and sterilize it. For calcified lesions, take sample images of the tissue containing the lesions to check the extent of calcification.**

*** Have patients wear a compression bandage for 2-3 days to prevent bleeding.**

●**Collecting subjective pain evaluation forms**

Patients will fill out subjective pain evaluation forms immediately after biopsy, one week after biopsy, and during the last patient follow-up. Patients will be asked to record their pain in numbers ranging from 0 for no pain to 10 unendurable pain.

● **Histological analysis, patient follow-up**

Vacuum-assisted biopsy results will be used as the final results. If additional breast surgeries are performed following the biopsy, results that are closer to malignancy will be used. If biopsy results show benign lesions, patients will be checked for any interval changes through a postoperative follow-up.

5.5 Statistical analysis – Principles and Method

●We will use SPSS software to analyze the sensitivity and specificity of the biopsy, and histological underestimation.

6. Research Schedule

Research Schedule (Total length of the research period: 12 months)

Description \ Month	Research Period												Note
	1	2	3	4	5	6	7	8	9	10	11	12	
• Recruit participants	■	■	■	■	■	■							
• Collect clinical data including ultrasound and biopsy results	■	■	■	■	■	■	■	■					
• Conduct a questionnaire survey regarding complications and pain	■	■	■	■	■	■	■	■	■	■			
• Collect follow-up data	■	■	■	■	■	■	■	■	■	■			
• Write a research paper on statistical analyses of the collected data										■	■	■	

7. Collecting consent forms from the participants

Among patients who require a biopsy based on breast ultrasound results, those, for whom a vacuum-assisted biopsy is deemed beneficial (patients who are expected to have lower rates of false negatives or histological underestimation after a vacuum-assisted biopsy), and who carefully read the information on the consent form, will be explained about the research purpose, surgical procedure, and associated complications by clinicians, and then be asked to submit their consent forms.

8. Measures to protect patient privacy and ensure patient safety

8.1 Protection of patient privacy

Of all researchers involved in this study, only a single study coordinator will have access to patients' private information. In the image files that will be sent to other researchers (the header part of DICOM files), patients' private information will be anonymized by converting it to random numbers.

8.2 Measures to ensure patient safety

The breast ultrasound that will be performed on the participants in the present study is generally considered safe. The 14G vacuum-assisted breast biopsy using a 14G biopsy needle will be performed while the patient is under local anesthesia. Bleeding and pain will be treated with conservative treatment. If a patient experiences severe bleeding, the emergency department will immediately be contacted.

9. Measures to ensure research ethics

The present study will be conducted after consideration by the Institutional Review Board (IRB) of Seoul National University of Bundang Hospital in compliance with the Declaration of Helsinki. Participants will be explained about the purpose of this study through information papers (refer to the consent form). Only the participants who are fully aware of the study purpose and risks associated with the biopsy that will be performed during the study, and fill out a consent form (refer to the consent form) will be able to participate in the study. Participants can cease participation on their own accord at any time, and should be aware of their right to do so.

Ultrasound-guided interventions are widely performed and considered safe in Korea, and has been approved by the Food and Drug Administration (FDA) of the United States. However, upon observation of mild side effects, the biopsy will be ceased and appropriate measures will be taken by researchers in charge.

Consent form

Research Title: Evaluation of the effectiveness of a 14-guage vacuum-assisted breast biopsy

We kindly ask you to participate in this study. In this study, a 14-gauge ultrasound-guided breast biopsy will be performed. Although the diagnostic accuracy of needle breast biopsies is generally very high, they have drawbacks such as false negatives resulting from small sample size (lesions are diagnosed as benign in a biopsy, but is later revealed to be cancer during surgery or postoperative monitoring), and histological underestimation (lesions are diagnosed as atypical changes in a biopsy, but are later diagnosed as ductal carcinoma or invasive cancer). In these situations, additional procedures will be required due to changes taking place in the surgical site. In most cases, enough samples can be collected from breast lesions by performing a needle biopsy alone. However, for patients with calcified lesions, intraductal lesions, or lesions less than 2.0cm in size, like yourself, there is a 10% chance of having false negatives or histological underestimation. The 14G vacuum-powered biopsy needle that is similar in thickness to the 14G needle commonly used in breast biopsies, but can collect larger amounts of tissues, is a new tool that overcomes all of the aforementioned drawbacks. Because it can collect twice as many samples during a single biopsy than regular biopsy needles, it will enhance the accuracy of the biopsy. In addition, a biopsy can be performed without multiple needle insertions when using the vacuum-powered needle. Since its thickness is similar to that of regular needles, it will leave scars of similar size to ones created by regular needles. These vacuum-powered needles that are of similar size to regular needles, but can collect larger amounts of tissues will be used in this study. Except for the breast biopsy and an examination in the department of pathology, patients will not be required to pay for any additional procedures. The vacuum-assisted biopsy will clarify things that were previously unclear in a needle biopsy.

The biopsy used in this study will follow the same procedure as the breast biopsy, and will use the principles of the Mammotome biopsy, which is a type of vacuum-assisted breast biopsy. A needle that is smaller than Mammotome will be used. However, the procedure may have a higher risk for bleeding than needle biopsies since larger amounts of tissues are collected, and may require additional 10 minutes for hemostatic treatment. Following the biopsy, you must answer simple questions related to the level of pain you experience. Please let us know if you experience any side effects due to local anesthetics. Since needles will be inserted during the biopsy, let us know prior to the procedure if you have previously experienced unstoppable bleeding or if you are on antiplatelet medications such as aspirin. You must cease taking antiplatelet medications for one week before undergoing a biopsy. If you are experiencing severe pain, you will be provided with analgesics. If you develop a hematoma that is greater than 5cm in size, follow-up ultrasonography will be performed free of charge to remove the hematoma.

All research data will be stored as secret codes; hence, only our researchers have access to your name. Your name will not be disclosed in any other studies. You may refuse to participate in the study, and can cease

participation at any time during the study period. You will not suffer any disadvantage related to your relationships with our doctors or the hospital as a result of refusing to participate or ceasing participation.

If you are not familiar or unsure about the meaning of the technical terms used in this consent form, please feel free to ask us. Please decide whether you would like to participate in this study or not after carefully reading the consent form. All participants will receive the first copy of consent form signed by them.

Consent: I, (Participant name) ______________ have read the consent form and have decided to participate in this study. I was fully explained about the general purpose of the study, things I must do during the study, and possible risks and discomfort caused by the procedures involved in the study.

20 year month day

Participant (Name/Signature): (Stamp)

Research director (Name/Signature): (Stamp)

If you have any inquiries regarding the present study or your rights as a participant, or have experienced any damage related to this study, please contact our research director at 031-787-2896.

Compensation Regulations

1. Principles

1.1 The present rule applies to damage experienced by participants participating in the evaluation of the usefulness of 14-guauge vacuum-assisted breast biopsy.

1. 2. Participants will receive compensation for any damage caused by the 14-gauge vacuum-assisted breast biopsy.

1. 3. Participants with temporary pain or damage that can be easily treated are not eligible for compensation. Compensation will be given for continuous damage that can permanently cripple the participant.

2. Conditions for compensation

Participants are not eligible for compensation if their damage is verified to be not causally associated with the 14-gauge vacuum-assisted breast biopsy, or if they are suffering from complications (pain, hematoma, side effects of local anesthetics, etc.) that were predicted prior to the procedure.

Participants are not eligible for compensation for damage that can also occur during other types of imaging-guided biopsies, or for damage whose severity does not exceed the severity predicted by modern medicine prior to the biopsy.

Participants will not be compensated for damage resulting from their own carelessness.

3. Compensation evaluation

3.1. An appropriate amount of compensation should be determined based on the medical fees required to treat the damage, and the type, severity, and risk of recurrence of the damage.

3.2. Once legal liability is acknowledged, the Korean court will maintain the amount of compensation at similar rates used for other damages.

3.3 When there is a disagreement regarding the amount of compensation between a participant and researchers, the participant should seek opinions from an independent specialist chosen under the mutual agreement, and respect his/her opinions in the determination of an appropriate amount of compensation. The participant will be fully responsible for any expenses that arise during this process.

I pledge to abide by the compensation regulations described above by ensuring that participants do not become disadvantaged as a result of the procedure performed during this study, and by taking responsibility for any damage that occurs as a result of this study.

Research Director

Department of Radiology, Seoul National University Bundang Hospital

Sun Mi, Kim(stamp)
