# Supplementary material for: Ultrasound-guided cable-free 13-gauge vacuum-assisted biopsy of non-mass breast lesions
Source: PLoS One. 2017 Jun 19;12(6):e0179182. doi: 10.1371/journal.pone.0179182 (PMC5476256; doi:10.1371/journal.pone.0179182)
Supplement: S2 File — (PDF) [file pone.0179182.s004.pdf]

Protocol outline

연 구 제 목	(영문) Evaluation of the usefulness of 14G vacuum-assisted breast biopsy in suspicious breast disease<br>(한글) 14게이지 유방 진공보조흡인 생검의 유용성 평가
연 구 목 적	일반적으로 초음파 유도하 유방 생검술에 사용하는 14G 총생검과 비교하여 좀 더 간편하게 많은 조직을 얻을 수 있어 조직학적 저평가나 위음성률이 낮을 것으로 생각되는 14G 진공 보조 흡인 생검의 적절한 적응증과 장단점을 확인해 보고자 한다.
연 구 기 관	분당서울대학교병원 영상의학과
연구책임자	분당서울대학교병원 영상의학과 김선미
연 구 대 상	총 216명의 환자
연 구 기 간	승인일로부터 1년
연 구 방 법	유방초음파를 시행 후 조직검사가 필요하다고 진단된 사람들 중 일반적으로 진공보조흡인 생검의 이득이 있는 것으로 알려진 석회화가 포함된 병변, 관내병변, 2.0cm 미만의 병변을 가진 환자군 중 이 연구 참가에 동의한 사람들을 대상으로 하여 진단의 정확도, 합병증 발생율, 환자 및 시술자의 편이성 등을 비교 평가하여 14G 진공 보조 흡인 생검침의 적절한 적응증과 장단점을 확인해 본다.
기대효과 및 예상결과	14G 진공보조흡인 생검은 코어 생검과 비교하여 볼 때 조직학적 저평가, 위양성이 적게 날 것으로 생각되고 환자의 시술 후 불편감, 통증 등에 차이가 없다면, 이를 사용할 경우 환자가 겪게 될 불필요한 추가 수술을 줄이고 정확한 진단을 할 수 있게 되므로 병원과 의사에 대한 신뢰도를 높일 수 있을 것이다.

1. 연구 제목

영문: Evaluation of the usefulness of 14G vacuum-assisted breast biopsy in suspicious breast disease

한글: 14게이지 유방 진공보조흡인 생검의 유용성 평가

2. 연구의 실시기관명 및 주소

분당서울대학교병원 영상의학과

경기도 성남시 분당구 구미로 173번길 82

3. 연구 책임자 및 담당자

3.1 연구책임자

분당서울대학교병원 영상의학과 부교수 김선미

3.2 연구담당자

분당서울대학교병원 영상의학과 조교수 윤보라

3.3 공동 연구자

분당서울대학교병원 영상의학과 조교수 장미정

분당서울대학교병원 영상의학과 전임의 안혜신

분당서울대학교병원 영상의학과 연구원 양은희

4. 예상연구기간

IRB 승인일로부터 1년

5. 연구방법

5.1 연구방법개요

1) 연구목적

일반적으로 초음파 유도하 유방 생검술에 사용하는 14G 총생검과 비교하여 좀 더 간편하게 많은 조직을 얻을 수 있어 조직학적 저평가나 위음성률이 낮을 것으로

생각되는 14G 진공 보조 흡인 생검의 진단의 정확도, 합병증 발생율, 환자 및 시술자의 편이성 등을 평가하여 14G 진공 보조 흡인 생검 침의 적절한 적응증과 장단점을 확인해 보고자 한다.

2) 연구배경 및 필요성

1. 초음파 유도 하 생검술

유방촬영술 또는 유방 초음파 검사에서 발견되는 비 촉지성 병변은 결절이나 미세 석회화 소견을 보인다. 영상만으로는 유방암과 양성 질환을 구별하기 어려울 때 생검술을 시행하게 된다. 초음파 유도 하 생검술은 외과적 생검술에 비해 덜 침습적이며 95% 정도의 높은 진단 정확도를 보인다. 유방촬영 유도 하 생검술에 비해 방사선 노출이 없고 환자가 편안한 자세로 누워서 시술을 받을 수 있으며 실시간으로 바늘의 위치를 확인할 수 있고 시술시간이 짧은 등의 여러 장점을 가지고 있다.

2. 14G 초음파 유도 하 총생검술의 위음성 진단과 조직학적 저평가

앞에서 이야기한 초음파 유도 하 생검술의 중대한 약점인 위음성 진단은 생검에서 양성으로 진단된 병변이 이후 시행한 수술에서 암으로 진단된 경우를 말하며, 14G 총생검침을 이용한 경우 0~3.6%(mean 1.8%)로 보고되어 있다. 미세석회화 병변이나, 고형성과 액체성 물질이 섞여있는 종괴, 5mm 미만의 작은 종괴, 유두종 들의 경우는 일반적인 병변에 비해 위음성률이 특히 더 높은 것으로 알려져 있다. 또한 생검에서 비정형 관상피 증식증으로 진단된 병변이 최종 수술 후 유방암으로 진단되거나, 생검에서 관상피 내암으로 진단된 병변이 최종 수술후 침윤성 유방암으로 진단되는 것을 말하는 조직학적 저평가 역시 문제가 될 수 있다.

경피적 생검으로 진단된 비정형 관상피 증식증의 경우 이환된 병변의 범위를 기준으로 조직학적 진단을 하는데, 한 유선관내에 존재하는 일반적인 상피세포 증식과 동반된 관상피 내암 소견의 크기가 2mm 이하이면 비정형 관상피 증식증, 2mm 이상이면 관상피 내암으로 정하였다. 따라서 병변의 일부에서만 비정형 또는 침습소견을 보이면 채취된 조직에 따라 저평가를 하게 될 수 있다.

3. 진공보조흡인 생검의 유용성

진공보조흡인 생검은 진공흡입기의 원리를 이용한 장비를 이용하여 병변의 조직을 얻는 방법이다. 침을 조직에 넣은 뒤 진공흡입기를 이용하여 조직을 침 안쪽으로 흡인하고 자르는 관으로 절단하여 절단된 조직을 진공 음압으로 자동으로

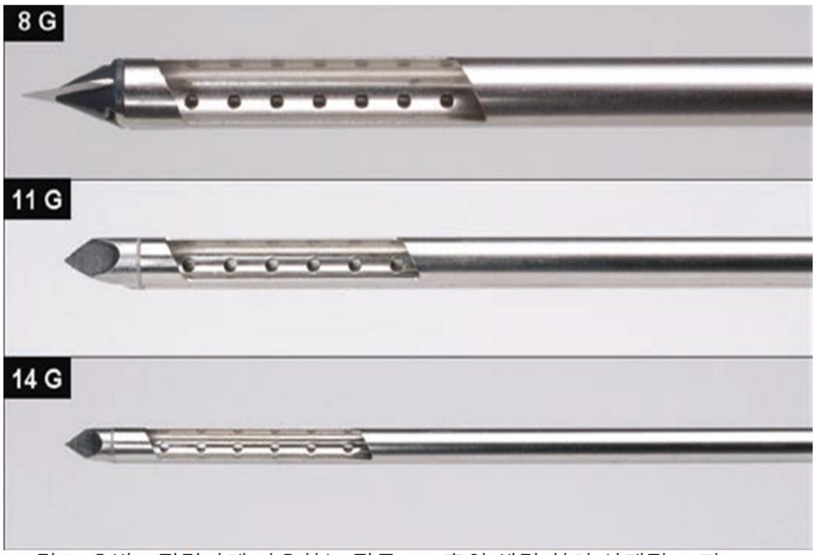

그림 1. 유방조직검사에 사용하는 진공보조흡인 생검 침의 상대적 크기

침 뒤쪽의 수집 공간으로 이동시켜 조직을 채취한다. 진공보조흡인 생검의 경우 총생검과 비교하여 조직 채취를 위해 침을 쏠 필요가 없으며, 한번만 종괴에 침을 넣으면 다시 유방 밖으로 침을 뺄 필요 없이 15-20회 정도 반복해서 쉽게 조직을 얻을 수 있다. 이로써 진단율은 높이고 재생검의 빈도를 줄일 수 있다. 사용되는 침의 굵기는 14G, 11G, 8G로 11G 침을 사용한 경우 민감도가 98-100%

특이도가 100%의 진단율을 보여 주었고, 침생검에 비해 비정형 관상피 증식증, 관상피 내암의 조직학적 저평가율이 낮다고 한다. 그 이유는 일반적인 14G 코어 침의 경우 한번 조직검사를 시행했을 때 17mg 의 조직을 얻을 수 있는 것에 비해, 8G, 11G, 14G 진공보조흡인 생검침은 각각 300mg, 100mg, 34mg 의 조직을 채취할 수 있기 때문인데, 즉 같은 gauge 를 사용해도 1회당 2배의 조직을 얻을 수 있다. 비정형 관상피 증식증의 11G 진공보조흡인 생검침의 저평가율은 16% (95% CI 12-20%)이고 관상피 내암의 경우 11% (95% CI 9-12%) 정도이다. 이에 비하여 비정형 관상피 증식증의 경우 총생검에서 40% (95% CI 26-56%), 관상피 내암의 경우 15% (95% CI 8-26%) 의 높은 조직학적 저평가의 발생률을 보였다. 그러나 14G 진공보조흡인 생검침의 경우는 같은 14G의 코어 생검침과 조직학적 진단성능이나 시술 후 부작용, 시술 후 불편감의 정도를 비교한 자료가 전무한 실정이다.

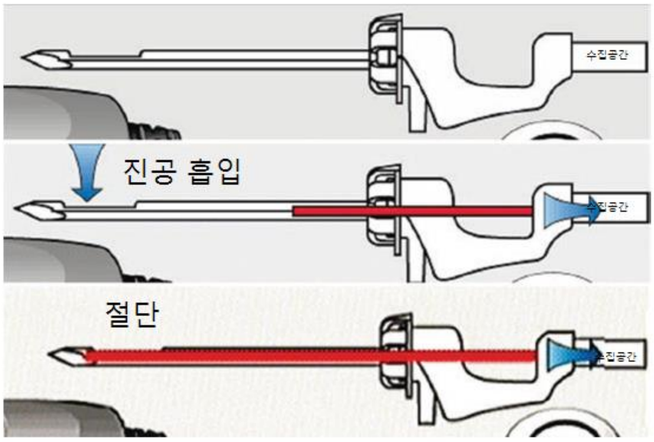

그림 2. 진공보조흡인 생검 침의 조직 채취법

5.2 피험자의 선정기준, 제외기준

● 선정기준: 유방초음파를 시행 후 조직검사가 필요하다고 진단된 사람들 중 진공보조흡인 생검의 이득이 있을 것으로 생각되는 환자군 중 이 연구 참가에 동의

한 사람들, 진공 보조 흡인 생검을 하면 위양성률이나 조직학적 저평가가 줄어들 것으로 생각되는 환자군(석회화가 포함된 병변, 관내병변, 2.0cm 미만의 병변, 고형성부분과 액체성 부분이 같이 있는 병변)

● 제외기준: 조직검사의 부적응증 (국소 마취제에 대한 부작용을 이전에 경험한 환자나 출혈 위험성이 높은 환자) 또는 연구 참가를 거부하는 환자는 제외

5.3 목표 피험자의 수 및 산출 근거

● 연구와 비슷한 이전의 선행 연구는 없었으나, 유두종의 11G 진공보조 흡인생검술의 조직학적 저평가에 대한 전향적 연구와 석회화의 11G 진공보조 흡인술에 대한 민감도와 특이도를 구한 전향적 연구가 있어 이 연구들을 인용하여 환자수를 산출하였다. 본 연구의 경우 pilot study 의 성격으로 검사의 민감도, 특이도, 조직학적 저평가의 비율과 부작용의 rate를 알고자 하는 것이다. 검정력 80%, 유의수준 5%로, 탈락률 5% 고려하여 216명의 환자가 산출되었다.

5.4 관찰항목, 관찰검사방법 및 임상검사항목

● 영상소견 획득

조직 검사를 하기 전 실시간으로 초음파를 보고 병변을 미국영상의학회 BI-RADS(Breast Imaging Reporting and Data system) 기준에 따라 description 함.

● 초음파 유도하 진공보조흡인 조직검사 방법

A) 검사방법

* 병변 부위 및 침이 들어갈 피부를 소독한 후 구멍포를 덮는다

*** 1% 리도카인 1-2ml 로 피부를 마취한다**

*** 11번 블레이드로 피부절개를 넣는다**

***1%리도카인 4-5ml 로 병변내 주위 침이 들어갈 경로를 마취한다.**

*피부의 절개를 통해 침을 유방내로 삽입한 후 초음파를 보며 병변 가까 이 위치시킴

*조직검사를 시행함

*시술 끝나고 출혈 방지를 위해 5-20분간 압박 후 피부 상처부위를 소독 함, 만약 석회화를 포함한 병변이라면 얻어진 조직에 대해 표본촬영술을 하여 석회화를 충분히 얻었는지 확인

* 환자에게 압박붕대를 2-3일 간 착용시켜 출혈을 방지한다.

● 환자 주관적 통증 평가표 획득

환자의 통증을 평가하기 위해 모든 환자는 주관적 통증 평가표를 시술 후 즉시, 시술 후 1주일, 마지막으로 시행한 추적 검사에서 작성 하도록 한다. 통증평가 척도는 0(통증 없음)에서 10(참을 수 없는 생애 최악의 통증)까지 사이의 숫자를 기록하게 한다.

● 조직학적 분석, 추적관찰

환자의 진공보조흡인 생검 결과를 최종 결과로 하되, 만약 추가로 시행한 유방수술이 있다면 둘 중 좀더 malignancy에 가까운 결과를 최종 결과로 함. 만약 조직검사에서 양성병변이 나왔다면 추적검사를 통해 interval change여부를 확인함.

5.5 통계분석 원칙 및 방법

● SPSS software사용, 검사의 민감도, 특이도, 조직학적 저평가를 통계로 분석한다.

6. 연구수행일정표

연구수행일정계획 (총 연구 기간: 12개월)

내용 월	연구기간												비고
	1	2	3	4	5	6	7	8	9	10	11	12	
• 대상 환자군 모집	■	■	■	■	■	■							
• 초음파, 조직검사 수술 결과를 포함한 임상 data 수집	■	■	■	■	■	■	■	■					
• complication과 통증 설문지 조사	■	■	■	■	■	■	■	■	■	■			
• 추적관찰 data 수집	■	■	■	■	■	■	■	■	■	■			
• 통계처리 논문작성										■	■	■	

7. 피험자 동의서 취득

본 연구에서는 유방초음파를 시행 후 조직검사가 필요하다고 진단된 사람들 중 진공보조흡인 생검의 이득이 있을 것으로 생각되는 환자군(진공보조흡인 생검을 하면 위양성률이나 조직학적 저평가가 줄어들 것으로 생각되는 환자군) 중 검사 시 피험자 동의서의 세부내용을 읽고 이 연구 참가에 응한 사람들에게 시술자가 검사목적, 시술과정, 합병증을 설명 후 동의서를 얻을 것임.

8. 피험자 사적 정보 및 안전 보호에 관한 대책

8.1 피험자 사적 정보 보호

연구에 참여하는 연구자 중 한명만 (study coordinator) 환자 개인 신상 정보에 접근할 수 있다. 다른 연구자들에게 주어지는 영상 파일에는 개인 신상 정보 (DICOM 파일의 Header 부분)을 난수로 변경하여 환자 정보를 익명화한다.

8.2 피험자 안전 보호에 관한 대책

연구에 참여하는 환자들에게 시행되는 초음파 검사는 일반적으로 알려진 대로 환자에게 안전하다. 시행되는 14게이지 진공흡인검사는 국소마취 하에 통증 없이 진행될 것이며 14게이지 생검 바늘을 사용할 것이다. 만일 출혈이나 통증이 야기되는 경우에도 일반적인 보존적 처치로 충분한 정도이며 심한 정도의 출혈의 경우 응급실로 즉각 연결한다.

9. 연구의 윤리성 확보를 위한 방안

본 연구는 헬싱키 선언에 따라 진행하며 분당 서울대 병원의 IRB (Institutional review board)의 심의를 받은 후 진행된다. 연구의 목적 특성은 피험자에게 설명문을 통해 설명 (동의서 참조)하며, 본 시험의 목적 및 위험 등을 알고 동의서 (동의서 참조)를 작성한 지원자만 시험에 참여할 수 있다. 지원자는 시험 중 언제라도 자유의사에 의해 시험을 그만둘 수 있으며 또한 이를 알고 있다.

초음파 영상 유도 중재적 시술은 우리나라에서 널리 시행되고 있고 안전한 검사로 알려져 있으며 미 FDA에 공인이 난 검사법이다. 그러나 경미한 부작용이라도 나타날 경우, 검사를 중단하고 반드시 책임 연구자가 적절한 조치를 취하도록 한다.

동 의 서

연구 제목: 14게이지 유방 진공보조흡인 생검의 유용성 평가

본 연구에 참여해 주실 것을 부탁드립니다. 본 연구는 초음파 유도하 14게이지 유방 진공보조 흡인 유방조직검사입니다. 일반적으로 유방 총생검의 정확도는 상당히 높은 편이지만, 가장 큰 약점은 부족한 샘플 양으로 인한 위음성 (조직검사에서 양성으로 나왔지만 수술이나 추적검사 중 암으로 밝혀지는 경우)과 조직학적 저평가 (조직검사에서 비정형성 변화로 나왔다가 최종 수술결과에서 상피내암이나 침윤성암이 나오는 경우 혹은 조직검사에서 상피내암으로 나왔는데 최종 결과에서 침윤성암으로 나오는 경우)입니다. 이럴 경우 수술 범위의 변화로 추가 수술이 필요하게 됩니다. 대부분의 유방 병변은 총조직 검사만으로 충분한 샘플을 얻을 수 있지만 환자 분 과 같은 석회화가 포함된 병변, 관내병변, 2.0cm 미만의 병변들의 경우 약 10%에서 위음성이나 조직학적 저평가가 있을 수 있는 것으로 알려져 있습니다. 유방조직검사에서 가장 흔히 사용하는 14G 총생검침과 비슷한 굵기의 바늘을 넣어 더 많은 조직을 얻을 수 있는 14G 진공보조흡인 생검침의 경우 이런 약점을 충분히 극복해 줄 수 있는 새로운 기구입니다. 조직검사에서 나오는 1개의 샘플양이 2배이기 때문에 좀 더 정확한 검사가 가능할 것으로 생각됩니다. 또한 여러번 바늘을 삽입하지 않고도 검사가 이루어 집니다. 바늘 굵기도 비슷하기 때문에 피부에 남는 흉터도 유사합니다. 즉, 조직검사를 시행하되 비슷한 굵기의 좀 더 조직을 많이 얻을 수 있는 바늘로 하는 것입니다. 일반적으로 시행하는 유방 총 조직검사의 비용과 병리과의 판독비를 제외한 추가되는 조직검사 시술비에 대해 환자분께 가해지는 경제적 부담은 없습니다. 오히려 총조직 검사에서 애매했던 부분이 진공흡인검사를 통해 명확해 질 수 있을 것입니다. 조직 검사 방법은 유방 조직 검사 시 받으실 것과 동일한 절차이며 이는 일반적으로 맘모톰이라

불리는 진공흡인유방조직검사와 같은 원리로 맘모톰보다 작은 바늘을 사용합니다. 단지, 유방 총조직검사와 비교해 많은 조직을 채취하므로 출혈의 가능성이 총조직 검사보다 다소 높을 수 있어, 검사 사 후 지혈 시간이 10분정도 더 걸릴 수 있습니다. 검사 후 통증 정도에 대한 간단한 답변을 해주셔야 합니다. 또한 국소 마취제를 사용하므로 약물의 부작용 경험이 있으셨다면 미리 얘기해 주십시오. 바늘을 삽입하는 검사이므로 출혈이 잘 멈추지 않은 경험이 있으시거나 아스피린 등 피가 잘 응고되지 않게 하는 약을 드시는 경우는 미리 알려주시고 1주일간 그 약을 드시면 안 됩니다. 만일 통증이 심할 경우 진통제를 제공받으실 것이며 혈종이 5cm이상 심하게 생기는 경우는 추적 초음파 검사를 검사비 없이 시행하여 혈종이 감소됨을 확인하여 드립니다.

모든 연구 기록은 비밀 번호로 관리되어 귀하의 이름은 연구자만이 알 수 있습니다. 연구 결과에 대한 어떤 학술 보고서에도 귀하의 이름은 밝히지 않을 것입니다.

귀하께서 연구에 참여를 거절하셔도 괜찮고 연구에 참여하신 후라도 언제든지 취소를 하실 수가 있습니다. 만일 귀하께서 거절하거나 취소하시더라도 저희 의사나 병원과의 관계에서 조금의 불이익도 받지 않을 것입니다.

저희가 이 동의서에 몇몇 전문 용어를 쓰게 되었는데, 혹시 이해가 잘 안 되는 것이 있으면 주저하지 말고 질문하여 주시고, 이 연구와 동의서에 대하여 충분히 검토하신 후에 연구 참여 여부를 결정하여 주시기 바랍니다. 또한, 서명된 동의서 사본 1부는 피험자에게 교부합니다.

승인: (피험자의 성명) _____________은/는 이 동의서를 읽고 위에 쓴 연구에 참여하기로 결정하였습니다. 이 연구의 일반적 목적, 해야 할 일, 있을 수 있는 위험성과 불편에 대하여 충분히 설명을 들었습니다.

20 년 월 일

피험자 (성명/서명): (인)

연구책임자(성명/서명): (인)

만일 귀하께서 이 연구나 피험자로서 귀하의 권리에 관하여 문의하실 것이 있거나 또는 연구와 관련하여 손상을 입으셨을 경우에는 연구 책임자 (031-787-2896)에게 연락하여 주십시오.

피해보상에 관한 규약

1. 원칙

1.1 본 규약은 14게이지 유방 진공보조흡인 생검의 유용성 평가에 참여하는 피험자에게서 발생한 상해에 대하여 적용된다.

1. 2. 손상의 원인이 14게이지 유방 진공보조흡인 생검으로 인해 발생하였을 때 환자에게 보상한다.

1. 3. 일시적 통증 또는 쉽게 치료될 수 있는 정도의 손상이 아니라 지속적이고 불구가 될 수 있을 정도의 보다 심각한 손상에 대해서만 보상한다.

2. **보상의 제한**

14게이지 유방 진공보조흡인 생검과 인과관계가 없는 상해임이 입증된 경우나 예상되는 합병증-동통, 혈종, 국소 마취제 부작용-인 경우 보상에서 제외된다.

다른 영상 유도하 조직 검사 하에서도 발생 가능한 정도를 넘지 않는 상해, 또는 상해가 현재의 의학적 지식으로 예견되는 정도를 넘지 않는 상해는 보상에서 제외된다.

환자의 부주의에서 초래된 손상에 대하여 대하여는 보상하지 아니한다.

3. 보상의 평가

3.1. 보상액은 해당 상해에 대한 의료비를 포함하여 상해의 종류, 중증도, 지속성에 따라 적절하게 산정되어야 한다.

3.2. 법적 책임이 인정되는 경우 한국법정이 유사한 상해에 대하여 통상적으로 지급하는 배상액과 일관된 수준을 유지한다.

3.3 보상수준에 대하여 의견 차이가 있는 경우, 의뢰자는 서로가 동의하는 독립전문가의 견해를 구하고, 양측은 적절한 보상수준을 결정함에 있어서 동 전문가의 견해를 존중하도록 한다. 의뢰자는 이에 따른 비용을 전액 부담하도록 한다.

본인은 앞에서 언급한 여러 제반 내용을 참고하여, 환자가 본 시험에 의해 어떠한 불이익도 받지 않도록 주의하며, 만약 본 시험에 의해 문제점이 발생할 경우 피해자 보상규약에 의거하여 책임을 질 것을 서약합니다

연구 책임자

분당서울대병원 영상의학과

김선미(인)
